# Local control and leptomeningeal disease after resection and GammaTile brachytherapy for newly diagnosed brain metastases: results from a prospective registry

**DOI:** 10.1007/s11060-026-05455-0

**Published:** 2026-02-07

**Authors:** Trent Kite, Simon Hanft, Sabrina Zeller, Stuart Lee, M. Sean Peach, Lindsey Sloan, Clark C. Chen, Vincent DiNapoli, Parag Sevak, Colette J. Shen, Rupesh Kotecha, Michael A. Garcia, David Brachman, Sita Patel, Adam Robin, Ian Lee, Huong Pham, Robert Ryan, William H. Smith, Andrea Wasilewski, Daniel Pavord, Rodney E. Wegner, Eugene C. Poggio, Matthew J. Shepard

**Affiliations:** 1 Department of Neurosurgery, Allegheny Health Network Neuroscience Institute, 320 E North Ave, Pittsburgh, PA 15212 USA; 2 Department of Neurosurgery, Westchester Medical Center, Valhalla, NY USA; 3 Department of Neurosurgery, ECU Health, Greenville, NC USA; 4 Department of Radiation Oncology, Brody School of Medicine, ECU Health, Greenville, NC USA; 5 Department of Radiation Oncology, University of Minnesota, Minneapolis, MN USA; 6 Department of Neurosurgery, Brown University Health, Providence, RI USA; 7 Department of Neurosurgery, Mayfield Brain and Spine, Cincinnati, OH USA; 8 Department of Radiation Oncology, Norton Healthcare, Louisville, KY USA; 9 Department of Radiation Oncology, University of North Carolina, Chapel Hill, NC USA; 10 Department of Radiation Oncology, Miami Cancer Institute, Baptist Health South Florida, Miami, FL USA; 11 GT Medical Technologies, Inc., Tempe, AZ USA; 12 Department of Neurosurgery, Henry Ford Health, Detroit, MI USA; 13 Department of Radiation Oncology, Virginia Mason Medical Center, Seattle, WA USA; 14 Department of Neurosurgery, Virginia Mason Medical Center, Seattle, WA USA; 15 Department of Radiation Oncology, St Luke’s University Health Network, Bethlehem, PA USA; 16 Department of Neuroscience, Allina Health, Minneapolis, MN USA; 17 Division of Radiation Oncology, Allegheny Health Network Cancer Institute, Pittsburgh, PA USA; 18 Biostatistical Consulting Inc., Lexington, MA USA

**Keywords:** GammaTile, Surgically targeted radiotherapy (STaRT), Brain metastases, Radiotherapy

## Abstract

**Purpose:**

Local failure and leptomeningeal disease (LMD) are both poor outcomes that can occur after resection and post-operative radiosurgery for newly diagnosed brain metastases (BM). There is increasing utilization of collagen-embedded Cesium-131 brachytherapy (GammaTile^®^) as a method of providing immediate adjuvant radiation therapy. Post-operative LMD rates following GammaTile implantation for newly diagnosed BMs has yet to be reported. The objective was to evaluate the incidence of LMD rates, local control (LC), and survival following resection and GammaTile for newly diagnosed BMs.

**Methods:**

An ongoing, multicenter, prospective, observational Phase IV non-interventional registry (NCT0442738) was queried to analyze rates of LMD following surgical resection of newly diagnosed BMs. Following resection and GammaTile implantation, we evaluated LMD rates, LC, and overall survival (OS). The Kaplan-Meier method was used to analyze time-to-event outcomes.

**Results:**

Fifty-one patients with 55 BMs were analyzed. The median follow-up was 12.4 months. The majority of BMs were in the supratentorial brain (87.3%). Four patients (7.8%) experienced LMD, 3 pachymeningeal and 1 classical. The 3-, 6-, and 12-month LMD-free rates were 97.4%, 94.1%, and 88.5%, respectively. The 12-month LC was 92.3%, and the 12-month OS was 49.0% with a median OS of 11.0 months.

**Conclusions:**

In this prospective registry study, GammaTile at the time of resection of newly diagnosed BMs was associated with high rates of tumor control and modest rates of LMD. As the trial registry continues to accrue, further data will continue to shed light on variables associated with outcomes.

## Introduction

Brain metastases (BMs) are the most common central nervous system (CNS) neoplasms [1]. While primary stereotactic radiosurgery (SRS) has been transformative in treating patients with small or medium sized asymptomatic lesions, surgery followed by adjuvant radiosurgery is often necessary for larger and/or symptomatic BMs [2]. Multiple previous studies have demonstrated a risk of post-operative leptomeningeal disease (LMD) following surgical resection and post-operative SRS, especially for infratentorial BMs, tumors derived from primary breast cancer, those of large size, and with pre-operative dural abutment [2–6]. A recent meta-analysis of 2105 surgically treated patients across 13 studies reported a median post-operative LMD rate of 16.1% over a median follow up of 13.4 months [3, 5]. The development of LMD carries an extremely poor prognosis with median overall survival (OS) rates ranging between 1 and 4 months [1, 7, 8].

The development of LMD following BM resection is attributed to mechanical tissue disruption with subsequent dissemination/seeding of tumor cells within the cerebrospinal fluid [1, 3, 9]. Therefore, en bloc resection and/or pre-operative SRS have been postulated to reduce the need for post-operative SRS [3, 5, 10]. However, en bloc resection of large and/or discohesive BM is not always feasible and pre-operative SRS is not always logistically possible, such as when patients live far from a radiation treatment center, or the patient is anticipated to have significant delay to radiation due to a prolonged rehabilitation stay. Additionally, at present, no prospective randomized trial has demonstrated a benefit of pre-operative SRS to post-operative SRS; therefore, post-operative SRS remains the only radiotherapy approach supported by level 1 evidence [11].

GammaTile^Ⓡ^ (GT) (GT Medical Technologies, Inc., Tempe, AZ, USA) is a type of brachytherapy that utilizes collagen tile-embedded Cesium-131 (Cs-131) tiles to deliver a highly conformal radiation plan at the time of tumor excision [12]. While often used in the recurrent setting of BMs that have failed prior SRS, the use of upfront GT confers several theoretical advantages over post-operative SRS, including immediate post-operative radiation administration and a higher biologically equivalent dose (BED) at the surgical resection cavity compared to other radiosurgical platforms [12–14]. Indeed, the efficacy of post-operative SRS versus GT is being investigated in a randomized clinical trial (NCT04365374). As GT affords immediate radiotherapy to the surgical cavity following surgery, the objective of this study was to investigate whether this approach impacts the incidence of LMD following craniotomy for radiation-naïve BMs. Secondarily we sought to determine overall local control (LC) rates of radiation-naïve BMs following implantation of GT brachytherapy.

## Materials and methods

### (NCT04427384) registry

An ongoing, accruing multicenter, prospective, observational Phase IV non-interventional registry (NCT04427384) was queried for patients receiving GT at the time of craniotomy for excision of radiation-naïve BMs.

At the time of this report (October 2025), the registry has accrued over 500 patients enrolled from 43 institutions. After excluding patients with primary CNS malignancies (i.e., gliomas, meningiomas, atypical tumors), recurrent tumors, and those concurrently enrolled in a separate interventional clinical trial ( NCT04365374), we arrived at a final cohort of 55 newly diagnosed BMs in 51 patients for this analysis. Patients are eligible for enrollment in this registry when GT is utilized in their care. Data, including patient demographics, tumor histology, prior CNS-directed therapy, and pre-operative and post-operative imaging are collected prior to surgery and at 1, 3, 6, 9, 12, 18, and 24 months and every 6 months thereafter for up to 5 years of follow-up. Patients presented herein were enrolled in the registry between August 2021 and March 2025.

As a non-interventional observational registry, the decision to treat patients with GT was at the discretion of the treatment team, including neurosurgeon and radiation oncologist, at each treatment center.

### GT technique and dosimetry

**Fig. 1:**
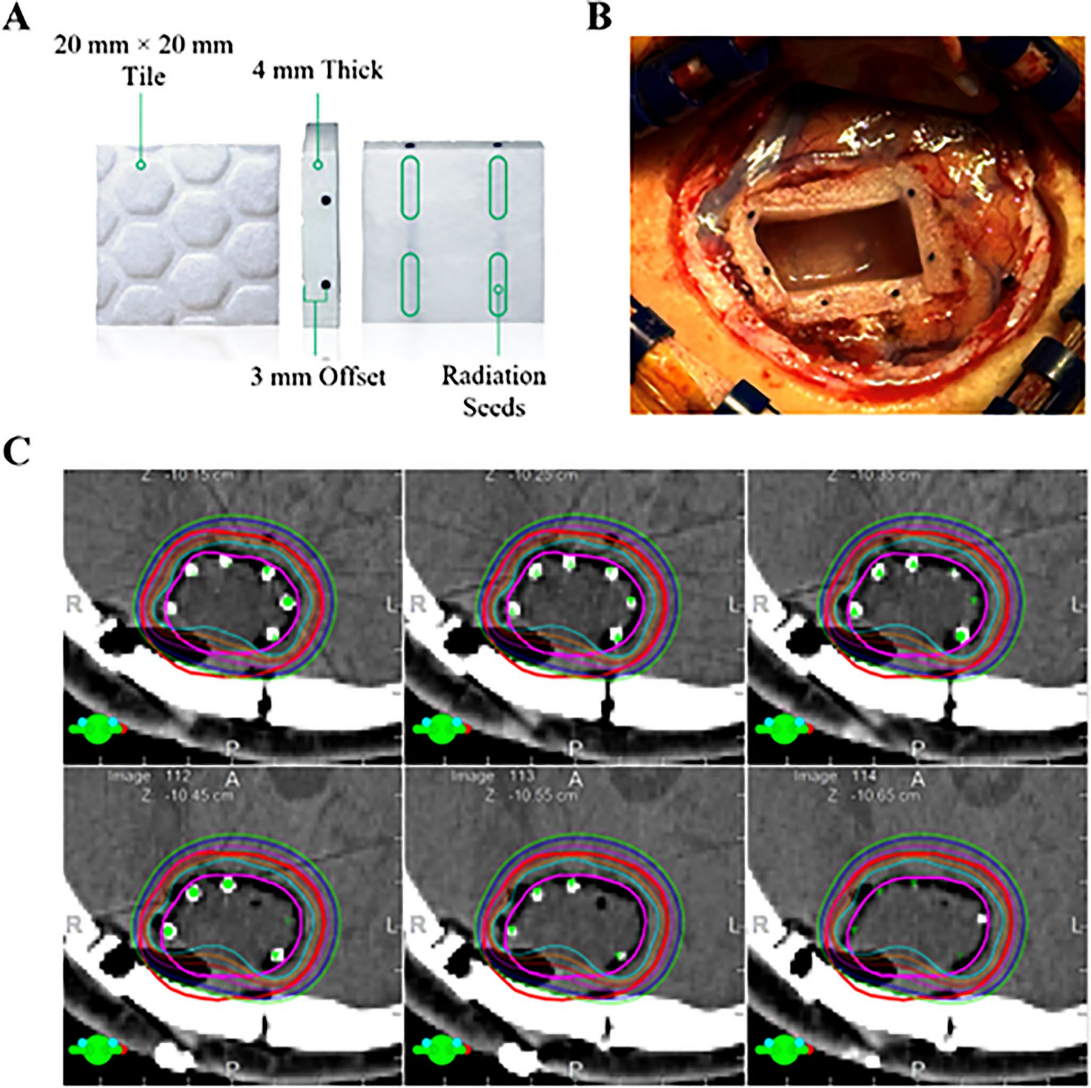
Overview of GammaTile device, placement, and dosimetry. (**A**) GammaTile is a 20 × 20 × 4 mm collagen tile with 4 cesium-131 radiation sources spaced symmetrically at 1 mm intervals, and asymmetric spacing of source depth with a nominal offset of 3 mm from the side with indentations versus 1 mm from the smooth side. (**B**) Intraoperative photograph demonstrating placement of 5 tiles in a post-operative resection cavity. (**C**) Post-operative dosimetry plan demonstrating highly conformal radiation plan

The technique of GT has been described previously [13, 15, 16]. In brief, after a standard craniotomy for BM resection has been completed, the resection cavity surface is lined with 20 mm × 20 mm × 4 mm collagen tiles that are embedded with Cs-131 seeds with individual source strengths of 3.5 U. The “bumpy” side of the tile is directed towards the brain surface when placed in the surgical cavity to ensure a 3 mm offset of the radiation source and brain parenchyma. The number of tiles needed for surgery is determined prior to surgery based on tumor size. Regarding dose, the BED at an α/β of 10 (BED_10 Gy_) at the cavity wall and at 2 mm depth is approximately 90 Gy and 57 Gy from GT, respectively (Fig. 1) [12].

### Data collection

Patients included herein were derived from treatment centers participating in the surgically targeted radiation therapy GammaTile registry (NCT04427384). Deidentified data including demographics (age, sex, and performance status), tumor characteristics (primary site, maximal diameter), procedure details (number of lesions and tiles implanted), receipt of concurrent systemic therapy at the time of GT implantation, defined as immunotherapy, targeted therapy, or chemotherapy ongoing at implantation or within one month before or after implantation, clinical outcomes (LMD, radiation toxicity, LC, distant tumor control, and OS) were determined by study site investigators and collected on the registry per study protocol. Adverse events (AEs) were graded in accordance with the Common Terminology Criteria for Adverse Events version 5 [44]. Data were de-identified and stored in a password-protected spreadsheet. All sites obtained patient consent prior to registry enrollment and gained approval from their respective institutional review boards (IRBs).

#### LMD criteria

Leptomeningeal disease (LMD) was prospectively collected via CRFs (case report forms) by querying whether a confirmed clinical diagnosis of LMD was present. When a confirmed diagnosis was reported, sites provided the date of diagnosis and classified the disease as either associated with/adjacent to the surgical bed or as a component of distant brain failure. Supplementary radiology reports and cytology reports were uploaded to the EDC (electronic data capture) when available. The diagnosis of LMD was largely based on serial imaging obtained within 1–3 month intervals following GT implantation.

### Statistical analysis

Continuous data were analyzed with descriptive statistics and reported as median (range/interquartile range [IQR])/mean [±standard deviation]) where appropriate. The Kaplan-Meier method was utilized to analyze time-to-event outcomes. OS was calculated from the time of GT implantation to the date of death, with patients censored on last clinical follow-up. Median clinical follow-up was determined using the reverse Kaplan-Meier method as previously described [17]. LC was analyzed on a per lesion basis using Response Assessment in Neuro-Oncology criteria [18]. LMD status was determined at the site level from follow-up imaging and further characterized into nodular meningeal or classical subtypes. Between group comparisons for time-to-event analyses were performed using the log-rank test. Between group analyses for categorical variables and continuous variables were performed using the Fisher’s exact test and the Mann-Whitney U test respectively. All statistical analysis was performed with Python (Version 3.12.7). The threshold for statistical significance was *p* < 0.05.

## Results

### Patient demographics

Fifty-one patients with 55 surgically excised lesions were eligible for analysis (Table 1). Among the 51 patients, a total of 132 BMs were present prior to surgery/GT implantation with a median of 1 (range: 1–16) BM per patient. The number of patients presenting with singular BMs and multiple BMs was 70.6% and 29.4% respectively. The cohort included 28 (54.9%) males and 23 (45.1%) females with a median age of 64 years (range: 28–81) (Table 1). At the time of GT implantation, the median Karnofsky Performance Status was 80 (IQR: 70–90). Primary tumor types included: non-small cell lung cancer (*n* = 21, 41.2%), melanoma (*n* = 8, 15.7%), breast (*n* = 6, 11.8%), renal cell carcinoma (*n* = 4, 7.8%), colorectal (*n* = 3, 5.9%), other (*n* = 8, 15.7%; see Table 1 for further breakdown), and unknown (*n* = 1, 2.0%). The median maximum pre-operative tumor diameter was 3.3 cm (1.1-5.4), and the median number of tiles implanted during surgery was 4 (range: 1–10).

**Table 1:** Pre-operative patient demographics and characteristics

Parameter	Detail
Number of patients, n	51
Age, years: median (range)	64 (28-81)
KPS at time of implantation: median (IQR)	80 (70-90)
Sex: n (%)	
Male	28 (54.9%)
Female	23 (45.1%)
Ethnicity: n (%)	
Non-Hispanic	48 (94.1%)
Hispanic	3 (5.9%)
Race: n (%)	
White	45 (88.2%)
Black/African American	6 (11.8%)
Primary malignancy: n (%)	
1. Lung (non-small cell)	21 (41.2%)
2. Melanoma	8 (15.7)
3. Breast	6 (11.8%)
4. Renal	4 (7.8%)
5. Colon	3 (5.9%)
6. Bladder	2 (3.9)
7. Esophagus	2 (3.9)
8. Ovary	2 (3.9)
9. Fibrosarcoma	1 (2.0%)
10. Mediastinum	1 (2.0%)
11. Unknown	1 (2.0%)
Symptomatic from BM at time of GT therapy n (%)	
1. Yes	48 (94.1%)
2. No	3 (5.9%)
Symptoms (%)	
1. Headaches	17 (33.3%)
2. Seizure	13 (25.5%)
3. Imbalance	13 (25.5%)
4. Aphasia	12 (23.5%)
5. Weakness/Paresthesia	11 (21.6%)
6. Confusion	10 (19.6%)
Concurrent systemic therapy at the time of GT implantation (%)	12 (23.5%)
Classification of systemic therapy (%)	
1. Immunotherapy	6 (50.0%)
2. Targeted therapy	3 (25.0%)
3. Chemotherapy	2 (16.7%)
4. Chemotherapy/Targeted therapy	1 (8.3%)
Concurrent systemic therapy per histology (%)	
1. Lung	6 (57.1%)
2. Melanoma	2 (25.0%)
3. Breast	1 (16.7%)
4. Fibrosarcoma	1 (100.0%)
5. Renal	1 (25.0%)
6. Ovarian	1 (50.0%)
Total number of surgically excised metastatic lesions: n	55
Total number of metastatic lesions: n	132
Median number of metastatic lesions per patient (range).	1 (1-16)
Supratentorial lesions:^a^ n (%)	48 (87.3%)
Gross total resection:^a^ n (%)	50 (90.9%)
Pre-operative maximum diameter (cm): median (range)	3.3 (1.1-5.4))
Number of Tiles used: median (range)	4 (1-10)

^*a*^Percentage expressed as a fraction of 55 lesions

*Abbreviations:* IQR = interquartile range; KPS = Karnofsky Performance Status; SD = standard deviation

Patients in this cohort were largely symptomatic at the time of surgery with 48/51 (94.1%) patients having at least one symptom from their BM. The most common reported symptoms were headaches (*n* = 17, 33.3%), seizures (*n* = 13, 25.5%), imbalance (*n* = 13, 25.5%), aphasia (*n* = 12, 23.5%), weakness/paresthesia (*n* = 11, 21.6%), and confusion (*n* = 10, 19.6%) (Table 1).

Patients included herein had not previously received whole brain radiation therapy (WBRT). Four (7.8%) patients underwent surgical excision of two BMs (and thus had two cavities implanted with GT). In total, 12 patients (23.5%) in the cohort were receiving concurrent systemic therapy at the time of GT implantation. Among these patients, immunotherapy was the most common modality (50.0%), followed by targeted therapy (25.0%), chemotherapy (16.7%), and combined chemotherapy and targeted therapy (8.3%). The systemic therapies administered included immunotherapy (pembrolizumab, nivolumab, atezolizumab), targeted therapy (alectinib, bevacizumab, everolimus), chemotherapy (pemetrexed/carboplatin), and combined chemotherapy and targeted therapy (entrectinib/doxorubicin). When stratified by primary tumor histology, the proportion of patients receiving concurrent systemic therapy was highest among those with fibrosarcoma (1/1, 100.0%), followed by lung cancer (6/21, 57.1%), ovarian cancer (1/2, 50.0%), melanoma (2/8, 25.0%), renal malignancies (1/4, 25.0%), and breast cancer (1/6, 16.7%). Gross total resection was achieved for 50 lesions (90.9%) in 47 patients (92.2%) during surgery as assessed by post-operative MRI (Table 1). The median clinical follow-up was 12.4 months (range: 1.0–33.6) (Table 2).

**Table 2:** Clinical outcomes following GT implantation

Parameter	Detail
Number of patients, n	51
Clinical follow-up (months): median (range)	12.4 (1.0-33.6)
LMD: n (%)	4 (7.8%)
LMD-free rates	
1. 3-months (%)	97.4%
2. 6-months (%)	94.1%
3. 12-months (%)	88.5%
12-month Local Control (%)	92.3%
12-month Overall Survival (%)	49.0%
Median Overall Survival: months	11.0

*Abbreviations*: GT = GammaTile; LMD = leptomeningeal disease

### LMD development

**Fig. 2:**
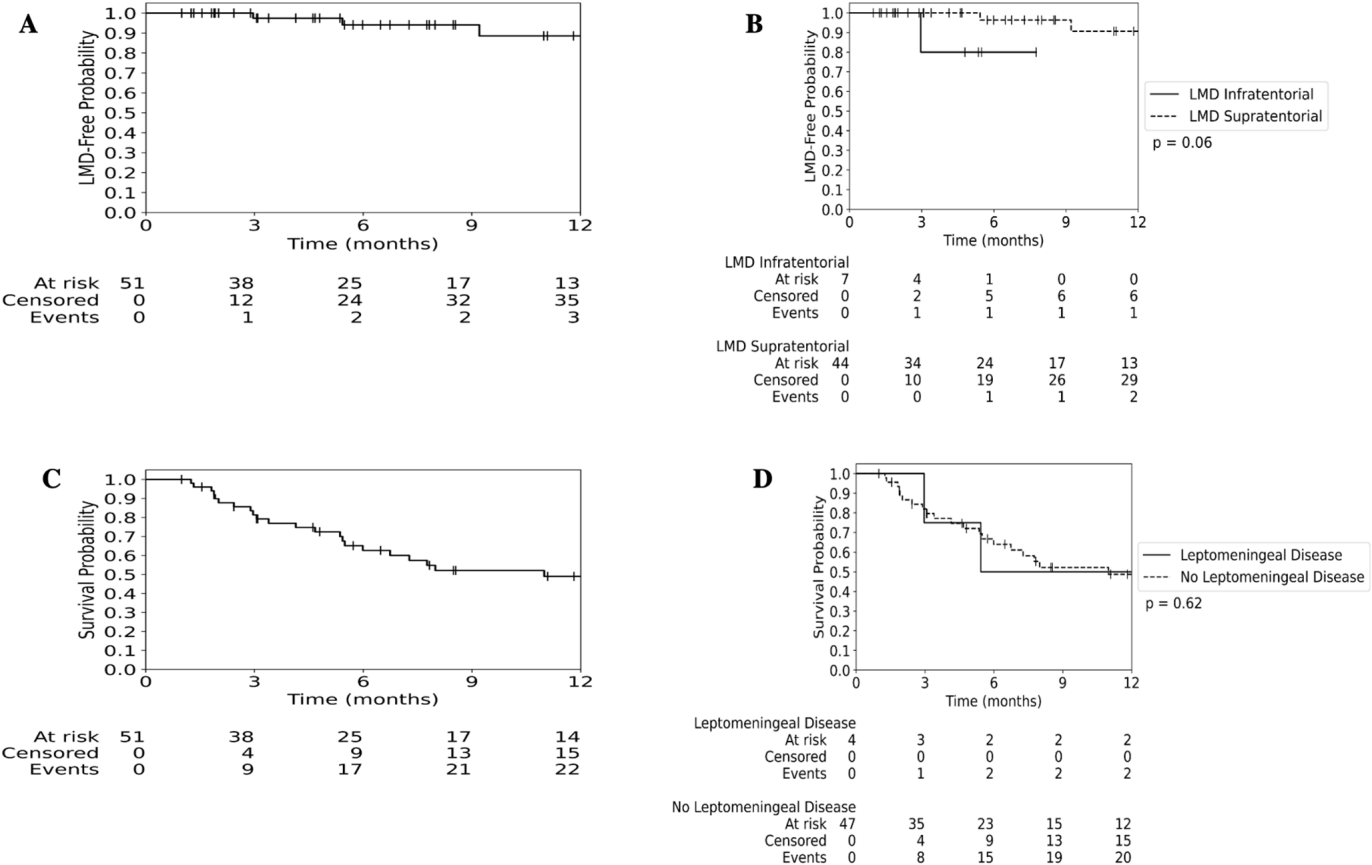
Resection plus GT demonstrates high LMD-free rates and encouraging overall survival. (**A**) Freedom from LMD over 12 months of follow-up. (**B**) Freedom from LMD stratified by supratentorial and infratentorial lesion location over 12 months of follow-up. (**C**) Overall survival of the entire cohort over 12 months of follow-up. (**D**) Overall survival stratified by LMD status over 12 months of follow-up

Following GT implantation, 4 (7.8%) patients reported LMD (Table 2) (Fig. 2A), of which 3 (75%) patients demonstrated a nodular meningeal LMD subtype while 1 (25%) demonstrated a classical LMD subtype. Cumulative LMD-free rates at 3-, 6-, and 12-months were 97.4% (95% confidence interval [CI]: 83.2–99.6%), 94.1% (95% CI: 78.0–98.5%), and 88.5% (95% CI: 66.7–96.4%), respectively (Fig. 2A and Table 2). For patients with infratentorial BMs, LMD-free rates at 3-, 6-, and 12-months were 80.0% (95% CI: 20.4–96.9%) at all timepoints, versus 100% (95% CI: 100–100%), 96.3% (95% CI: 76.5–99.5%), and 90.6% (95% CI: 66.5–97.7%), respectively, for those with supratentorial BMs (*p* = 0.06) (Fig. 2B). A univariate analysis comparing patients with LMD development versus those without LMD development is summarized in Table 3. After the development of LMD, the median OS was 5.4 months (Fig. 2D).

**Table 3:** Comparison of patients with LMD versus those without following resection plus GT

Parameter	LMD (n=4)	Non LMD (n=47)	p-value
Age (median)	66.0	64.0	0.54
Female, n (%)	2 (50%)	21 (44.7%)	>0.99
Active systemic therapy, n (%)	2 (50%)	10 (21.3%)	0.23
KPS >90, n (%)	3 (75%)	19 (40.4%)	0.30
Breast histology, n (%)	2 (50%)	4 (8.5%)	0.06
GTR, n (%)	3 (75%)	45 (95.7%)	0.22
Multiple brain metastases undergoing GT implantation, n (%)	0 (0%)	4 (8.5%)	>0.99
Infratentorial location, n (%)	1 (25%)	6 (12.8%)	0.46

*Abbreviations*: GT = GammaTile; GTR = gross total resection; KPS = Karnofsky Performance Status; LMD = leptomeningeal disease

### Overall survival and local control

**Fig. 3:**
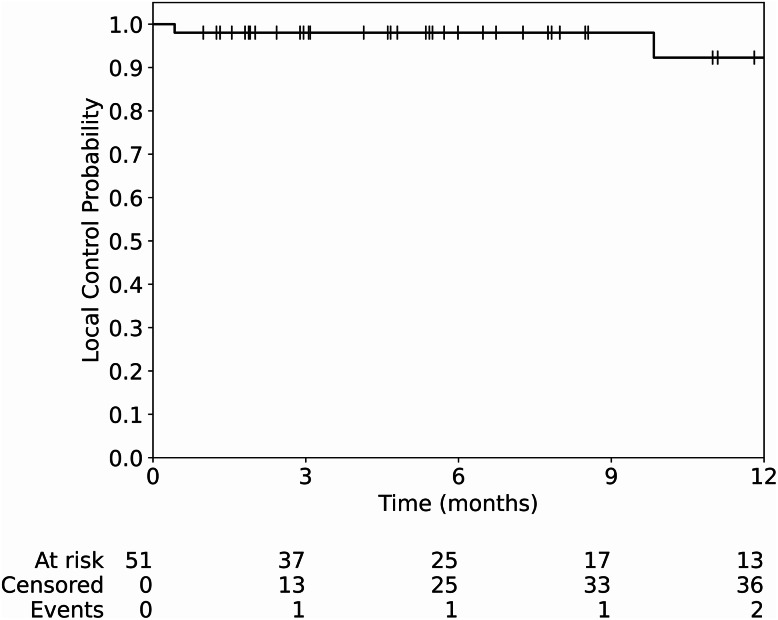
Resection plus GT demonstrates high rate of tumor local control over 12 months of follow-up

The median OS in this cohort was 11.0 months. The OS rates at 3-, 6-, and 12-months were 81.4% (95% CI: 67.2–89.8%), 62.6% (95% CI: 46.7–75.0%), and 49.0% (95% CI: 33.0–63.2%), respectively (Fig. 2C). The median OS for patients who developed LMD was 5.4 months versus 11 months for those without LMD (*p* = 0.62) (Fig. 2D). The LC rates at 3-, 6-, and 12-months were 98.0% (95% CI: 86.9–99.7%), 98.0% (95% CI: 86.9–99.7%), and 92.3% (95% CI: 68.3–98.3%), respectively (Fig. 3).

### Safety

Notable AEs per patient were as follows: two instances (*n* = 2, 3.9%) post-operative cerebral edema, two wound infections (*n* = 2, 3.9%), one of which necessitated surgical intervention, and one patient who developed new weakness post-operatively (1.9%). Overall Grade 3 toxicity was seen in 5 (9.8%) patients.

## Discussion

Surgical excision has been identified as a risk factor for tumor dissemination with subsequent LMD formation in BMs [1, 19, 20]. Rates of LMD following surgical excision alone have been reported up to 33% but are generally reported to occur in approximately 16.1% of patients following craniotomy [4, 21].

For patients undergoing resection of BMs, post-operative SRS has traditionally been employed to reduce the incidence of local recurrence but has not been associated with mitigating post-operative LMD formation [2]. In Mahajan et al.’s Phase III randomized controlled trial comparing post-operative SRS versus observation following craniotomy for BM excision, the 12-month LMD rate was 16% in the observation arm and 28% in the SRS arm [2]. Thus, while post-operative SRS may improve LC rates compared to observation alone, it does not appear to mitigate the risk of post-operative LMD development. Furthermore, LC rates of 72–84% with adjuvant SRS in the modern era are unacceptably low [2, 22], especially as survival has improved for patients with brain metastases due to improvements in systemic therapy.

Due to the rates of local failure as well as LMD after post-operative SRS, there is ongoing investigation and clinical trials examining the feasibility and utility of pre-operative SRS to “sterilize” the tumor prior to surgical excision to decrease the likelihood of post-operative LMD [9, 23, 24] While the mechanism of pre-operative tumor cell sterilization is not fully elucidated, it is believed to be secondary to cell cycle arrest with subsequent loss of migratory potential in active tumor cells present at the time of SRS [9, 10, 23, 24]. Several centers have examined the incidence of LMD rates following pre-operative SRS and have reported 12-month LMD rates ranging between 0% and 4.8% [24].

Our data are notable for a post-operative LMD rate of 7.8% with a 12-month freedom from LMD rate of 88.5% which appear superior to what has been previously described in the literature. Tewarie et al. recently published a meta-analysis examining the incidence of LMD following craniotomy for BMs [5]. In this study, 386 of 2105 examined patients (18.3%) developed post-operative LMD. Breast cancer, the presence of multiple BMs, and infratentorial location of the excised BM were risk factors for LMD development. Other authors have demonstrated that en bloc resection of BMs may decrease the incidence of post-operative LMD, although en bloc resection rates for supratentorial BMs may only be possible in 65% of intended cases [4]. Our results are consistent with other trends for post-operative LMD including breast cancer histology comprising 50% of LMD cases.

While several studies appear to demonstrate decreased rates of post-operative LMD with pre-operative SRS, this approach may not be generalizable to other treatment centers without access to inpatient radiosurgical platforms. Furthermore, the coordination of SRS and operating room availability creates a logistical barrier to pre-operative SRS. While post-operative SRS remains the standard of care, not all patients are able to receive timely adjuvant SRS. O’Brien et al. examined 159 patients referred for post-operative adjuvant SRS. In this report, only 25.2% of patients began SRS within 4 weeks of surgery. In one-third of their cohort, SRS was delayed by at least 2 months or never received [25]. Furthermore, rapid BM recurrence has been reported in some patients while awaiting adjuvant SRS [25, 26]. Strategies such as intraoperative brachytherapy and intraoperative radiotherapy (IORT) overcome many of these logistic concerns as radiation therapy is employed immediately at the time of craniotomy [27].

These data are also notable for a 12-month LC rate of 92.3% which exceeds the 72% 12-month LC rate described in radiosurgical arm of the Phase III randomized controlled trial comparing post-operative SRS versus observation following BM excision [2]. Our control rate is similar to the 95.4% 12-month LC rate described by Udovicich et al., who performed a multicenter study examining pre-operative SRS in 179 patients, and the 94% 12-month LC rate described by Cifarelli et al., who described the outcomes of IORT for BMs undergoing excision [24, 27]. Taken together, these findings suggest that immediate radiation at the time of surgery may provide improved LC and reduced LMD risk compared to post-operative SRS, while also addressing key logistical barriers to timely adjuvant treatment.

Contemporary literature supports the use of adjuvant SRS following resection of brain metastases (BMs) [2]. Although this approach has largely become standard practice, emerging evidence suggests that neoadjuvant (pre-operative) SRS may represent a viable alternative [12, 28–31]. Reported 1–2-year local control rates range from 77.2%–96.3% [12, 28–32].

In the present series, a 12-month local control rate of 92.3% was observed, suggesting that local control outcomes among pre-operative SRS and brachytherapy may be comparable. Increasing attention, however, has been directed toward differences in leptomeningeal disease (LMD) rates between treatment strategies. Multiple studies have reported lower LMD rates with pre-operative SRS (1.9%–4.3%) compared with post-operative SRS (0.0%–18.0%). Indeed, Yoo et al. most recently reported a significant reduction in LMD with pre-operative SRS (4.29% vs. 18.0%, *p* = 0.046) [32].

While the LMD rate reported herein compares favorably with historical post-operative SRS cohorts, the consistently low LMD rates reported with pre-operative SRS warrant consideration and will likely be an area of future investigation. At present, the literature supporting adjuvant post-operative SRS to the resection cavity is more mature than that for pre-operative SRS, justifying its role as the current standard comparator for GT implantation. Ongoing clinical trials, including NCT03750227 [33], may further refine these paradigms.

Ultimately, definitive conclusions will require direct comparison of all three strategies in large, prospective, multi-institutional studies. From a practical standpoint, the implementation of pre-operative SRS remains limited to centers with specific technical capabilities. In this context, GT implantation may serve as an important alternative when pre-operative SRS is not feasible.

### Limitations

This is a single-arm prospectively enrolled cohort study designed to be descriptive in nature. Consequently, these results require validation in larger, randomized cohorts. As the study was not designed to directly compare outcomes with patients who underwent WBRT or SRS with GT following surgical excision, conclusions based on comparison to historical data are circumstantial. Furthermore, given the limited occurrence of LMD cases, a Cox regression analysis could not be performed without a significant risk of model overfitting and spurious findings. We were therefore insufficiently powered to detect predictors of LMD. Furthermore, interpretation of the LMD point estimate across a limited cohort must be done with caution. Additionally, the influence of concurrent systemic therapy at the time of GT implantation on survival outcomes must be acknowledged. Previous studies have consistently demonstrated the positive relationship between systemic therapy and improved local control as well as survival outcomes in intracranial tumors undergoing radiosurgical intervention [34–40]. With approximately a quarter of the present cohort receiving concurrent systemic therapy, survival outcomes cannot be exclusively attributed to local control following GT implantation. Molecular features often inform the selection of systemic therapies, data which was not captured in the registry. Therefore, we are unable to assess the impact of molecular characteristics on outcomes in our cohort. Furthermore, greater than half of the cohort presented with a singular BM, indicating a relatively limited disease burden intracranially. As a result, outcomes in this cohort may be favorably skewed. We view these results to be hypothesis generating and the basis for future investigation. The efficacy of post-operative SRS versus GT is being investigated in the randomized clinical trial (NCT04365374)**.** Given the increasing popularity of neoadjuvant SRS in the management of BMs, similar trial designs may be necessary to evaluate the comparative efficacy of GT and pre-operative SRS. Although there is a theoretical concern that CT artifact from GT can adversely affect future treatment planning, MRI artifact secondary to GT implantation is negligible ("there are only tiny signal dropouts at the seeds). Given prior experience with I-125 brachytherapy, there have been concerns over possible increased radiation necrosis rates with brain brachytherapy. However, multiple independent institutional reports of collagen-embedded cesium-131 brachytherapy use for BMs, even in the re-irradiation setting, have not shown an elevated incidence of radiation necrosis, likely due to the steep-dose gradient properties of GT [14, 26, 40–43].

## Conclusion

Herein, we present a prelimary report from a multi-institutional prospective registry study to examine the rates of LC and LMD, along with safety, following surgical excision of newly diagnosed BMs treated with GT brachytherapy. The observed rates are favorable in the context of historical adjuvant SRS. Further data from this registry, along with randomized data, will support future analyses.

## Data Availability

Data is available upon reasonable request.
